# The Management of Acute Exacerbations in COPD: A Retrospective Observational Study and Clinical Audit

**DOI:** 10.3390/jcm13010019

**Published:** 2023-12-19

**Authors:** Maria Boesing, Nicola Ottensarendt, Giorgia Lüthi-Corridori, Jörg D. Leuppi

**Affiliations:** 1 University Institute of Internal Medicine, Cantonal Hospital Baselland, 4410 Liestal, Switzerland; 2 Faculty of Medicine, University of Basel, 4056 Basel, Switzerland

**Keywords:** COPD, exacerbation, GOLD guidelines, adherence, audit

## Abstract

(1) Background: Acute exacerbations of chronic obstructive pulmonary disease (COPD) are not only associated with increased patient morbidity and mortality, but with extensive healthcare costs. Thus, adequate clinical management is crucial. The aim of this project was to evaluate the management of acute COPD exacerbations in a public teaching hospital in Switzerland. (2) Methods: We retrospectively analyzed clinical routine data of patients presenting with an acute exacerbation of COPD at the emergency department of a Swiss hospital between January 2019 and February 2020. Management was evaluated against recommendations from the GOLD 2019 report and previous audits. (3) Results: The data of 184 patients (mean age 73.5 years, range 41–95 years, 53% male) with 226 visits were included. While the documentation of GOLD stage (I-IV) and smoking status was consistent (81.0% and 91.6%), GOLD risk category (A-D) was only documented in 36% of the cases. Patients’ respiratory rate upon presentation was measured in 73%, and blood gas analysis was performed in 70%. A total of 94% of the patients received a chest imaging; spirometry was performed in 10%. Initial symptomatic therapy with short acting bronchodilators was applied in 56%. Systemic steroid treatment was installed in 86%. Antibiotics were given in 56%, but in one fourth the indication was not clear. Non-invasive ventilation was applied in 25% of the indicated cases. Smoking cessation was recommended to 26% of the current smokers and referral to pulmonary rehabilitation was given in 16%. (4) Conclusion: GOLD recommendations were not comprehensively implemented, especially with regard to the assessment of severity, initial symptomatic therapy, and non-invasive ventilation. These results show the importance of the frequent revision of routine practice and may help to create awareness among practitioners and ultimately improve the quality of COPD management.

## 1. Introduction

Chronic obstructive pulmonary disease (COPD), a frequent, progressive and preventable disease, is a major global public health issue affecting approximately 5% of the worldwide population [1]. While it affects mainly smokers and ex-smokers, the risk of developing COPD is driven by several other risk factors [2,3]. COPD remains the third leading cause of death worldwide [1]. It is characterized by an irreversible airflow limitation, causing chronic dyspnea and cough, often with phlegm, and sometimes emphysema [4]. Acute exacerbations of COPD (AECOPD) are defined as an acute aggravation of the patient’s respiratory symptoms (dyspnea, cough, and/or sputum) that results in additional therapy [5]. They are classified as mild (treated with short acting bronchodilators only), moderate (treated additionally with antibiotics and/or oral corticosteroids), or severe (patient requires hospitalization or visits the emergency room) [5]. AECOPD are mostly triggered by viral or bacterial infection and are associated with increased morbidity and mortality [6,7]. Previous studies showed that between 25% and 50% of COPD patients experience at least one exacerbation per year that requires (additional) pharmacological treatment [8]. AECOPD constitute the main reason for the hospitalization of COPD patients and have a negative impact on the general disease progression [9,10]. The key objective of AECOPD treatment is to minimize these adverse effects [5].

Recommendations from the regular report of the Global Initiative for Chronic Obstructive Lung Disease (GOLD) are considered the basis for national and international guidelines regarding COPD management all over the world. Since the publication of the GOLD-report 2017, COPD patients are classified into groups (A,B,C,D), depending on symptom impact on daily life, and exacerbation frequency and severity. Current GOLD recommendations for COPD basis therapy are aimed at the reduction of symptoms and future risk of exacerbations [11]. They are based on the A-D classification and include short-acting (SABA) and long-acting beta-2 agonists (LABA), short-acting (SAMA) and long-acting antimuscarinic antagonists (LAMA), inhaled corticosteroids (ICS), and systemic corticosteroids, as well as non-pharmacological interventions [11].

In AECOPD, recommended diagnostic steps include the assessment of respiratory rate, arterial blood gas analysis, and chest imaging [11]. The main pillars of AECOPD treatment are oxygen supplementation, SABA, systemic corticosteroids, and, if indicated, antibiotics and/or (non-) invasive ventilation [4]. Further recommendations include an early follow-up, smoking cessation and pulmonary rehabilitation, physical activity, and vaccinations (pneumococcal, influenza) [11]. Previous audits of AECOPD management showed major deviations from recommended procedures [12,13,14]. The European COPD audit (2011) with the data of over 16, 000 patients revealed that only in 15.3% of the analyzed cases all ten investigated GOLD-10 recommendations for the management of AECOPD were implemented in clinical practice [15]. The Swiss results from this audit showed discrepancies, in particular, with regard to the availability of spirometry results (64%) and the performance of blood gas analysis (86% of indicated), as well as acute therapy with short-acting bronchodilators (79%) and non-invasive ventilation (NIV) (67% of indicated) [12]. However, because of the above-described implications of AECOPD, their adequate treatment is a crucial factor in the general management of the disease.

### Objectives

Clinical audits are fundamental for the evaluation and improvement of clinical processes [16,17]. The most recent Swiss data on this topic is over ten years old, and in the meantime, GOLD recommendations have been adapted several times. In this project, we aimed to assess the clinical management of AECOPD in a Swiss public teaching hospital, in order to evaluate the level of adherence to current GOLD recommendations in a narrative discussion. Ultimately, the results of this study will allow the generation of concrete and practical suggestions to improve the clinical management of AECOPD.

## 2. Materials and Methods

### 2.1. Study Design

This project was a retrospective, observational single-center study. Patients presenting with AECOPD at one of the three emergency department (ED) sites of the Cantonal Hospital Baselland, Switzerland (KSBL), and fulfilling the eligibility criteria (see Section 2.2) were included in the study. Due to potential bias caused by the SARS-CoV2 pandemic, the study period was chosen as January 2019 to February 2020 (pre-pandemic). In the case of patients’ recurring emergency presentation in the given period, each presentation was evaluated as a single case. The KSBL is a public teaching hospital providing medical care for a population of about 250,000.

### 2.2. Eligibility Criteria

Cases were included in the analysis if the following eligibility criteria were fulfilled:

- Presentation with AECOPD at the ED between January 2019 and February 2020;
- At least 18 years old;
- No written or verbal denial to the hospital’s general research consent;
- Confirmed diagnosis of COPD before admission;
- AECOPD was documented as the main diagnosis.

### 2.3. Data Collection and Management

Inpatient cases were pre-selected from the data of the hospital’s controlling department, filtering the main diagnosis for the ICD-10 codes J44.0 (Chronic obstructive pulmonary disease with acute lower respiratory infection) and J44.1 (Chronic obstructive pulmonary disease with acute exacerbation, unspecified) in the given period. Since outpatient cases were not routinely coded with ICD-10 codes, the daily overview schedules of all three ED sites were screened manually for patients presenting with AECOPD. After verification of the eligibility criteria, the required patient data were manually extracted from the digital patient files. These included discharge reports, nursing documentation, emergency reports, laboratory records, radiology findings, and spirometry results. Data were entered into a REDCap^®^ (Research Electronic Data Capture) database.

### 2.4. Guidelines

Relevant sections of the 2019 GOLD report, which was valid for the study period, were summarized and reduced to the most important recommendations for the management of AECOPD [5]. Each included case’s clinical management was evaluated with these recommendations, summarized in Table 1. The treatment with antibiotics was additionally considered as indicated, if a serum procalcitonin-based protocol had been used, as recommended in the Swiss national guidelines of 2018 [10].

### 2.5. Analytical Methods

Data were analyzed in a descriptive manner using REDCap^®^’s built-in analysis tools. Results are presented as absolute and relative frequencies, mean and standard deviation (SD), or median and interquartile range (IQR), unless otherwise stated. Associations between guideline adherence and patient outcomes were analyzed by means of the Mann–Whitney U test (length of hospital stay) and chi-squared test (in-hospital death) with R version 4.0.3.

### 2.6. Ethical Considerations

The ethics commission of Northwestern and Central Switzerland (EKNZ) approved this research project (BASEC-ID 2022–00217). An exception to the patients’ informed consent policy was granted in accordance with Article 34, Swiss human research law (Art. 34, HFG) by the EKNZ. Data of patients who had denied the hospital’s general research consent were excluded from the analysis.

## 3. Results

Case pre-selection resulted in 288 cases of AECOPD at the ED during the study period. After applying the eligibility criteria given in Section 2, 226 cases were included in the analyses. Twenty-seven patients presented twice, and six patients presented three times with AECOPD during the study period, resulting in 184 included patients overall. In the majority of the cases (*n* = 143, 63%), the presentation was initiated by the patient. The remaining cases were referrals by general practitioners, other medical specialists, or other hospitals. While 18 cases were managed as outpatient cases (8%), the majority of patients were hospitalized in the normal ward (*n* = 183, 81%). Four cases were managed in the intermediate care unit (1.8%) and twenty-one cases in the intensive care unit (9.3%). A flow chart of the case selection process and a summary of the cohort characteristics can be found in the Supplementary Materials.

The results presented in this section are limited to the direct evaluation of the implementation of the guidelines presented in Table 1. A more comprehensive assessment of performed procedures, prescribed therapy, and discharge planning can be found in the Supplementary Materials.

### 3.1. Anamnesis, Diagnostics and Documentation

Performed anamnesis, diagnostic procedures and documentation are presented in Table 2.

Although the knowledge of GOLD risk group (A–D) is crucial for discharge recommendations and base therapy decisions, it was only documented in 40% of the cases (*n* = 91). With regard to the assessment of AECOPD severity, the respiratory rate was documented in 73% (*n* = 164). The assessment of changes in mental status and use of respiratory muscles was only documented when present (*n* = 14 (6%) and *n* = 15 (7%), respectively), but not documented in a single case when not present. Oxygen saturation was assessed in nearly all cases (*n* = 220 (97%) and *n* = 223 (99%), respectively). The recommended arterial blood gas analysis (BGA) was performed in 62% of the cases (*n* = 141). Chest radiography was performed in 212 cases (94%) (chest x-ray 81%, chest CT 7%). In 54 cases (24%) the results of the last spirometry were documented.

### 3.2. Therapy

Table 3 summarizes the therapies applied to AECOPD patients in the ED and during hospitalization.

In the 48 patients with an oxygen saturation <88%, all but one was treated with supplemental oxygen (98%). While 64 cases presented an indication for invasive or non-invasive ventilation, only 17 of the affected patients were treated accordingly (fifteen with NIV (23%), two with MIV (3%)). The most common indication for NIV was severe dyspnea with documented signs of respiratory muscle fatigue or increased work of breathing (32 cases), followed by respiratory acidosis (30 cases). These two were also the most prevalent indications, in which NIV was not administered, although recommended (27 cases and 17 cases, respectively). Three patients received NIV with an unclear indication. MIV, on the other hand, was indicated in seven cases due to NIV failure (5 cases), cardiac arrest (1 case), and diminished consciousness with life-threatening hypoxemia (1 case). MIV was applied in the latter two cases, while the other five patients (or their relatives) declined further escalation of the therapy.

The recommended initial symptomatic therapy with SABA was applied in only half of the cases (*n* = 118, 52%). Systemic steroids, on the other hand, were administered in 89% of the cases (*n* = 200), with a median treatment duration of 5 days and a median daily dosage of 40 mg prednisone equivalent. While in nine cases systemic corticosteroids were administered for longer than seven days, in 95% of the cases they were given guideline-adherent for no longer than seven days (*n* = 180). In three quarters of the cases corticosteroids were dosed as recommended with 40 mg prednisone equivalent (*n* = 143, 76%). Antibiotics were administered in 127 cases (56%), while 27 of those (21%) did not present a clear indication. On the other hand, 16 patients did not receive antibiotics, even though 116 had a clear indication for it (14%). The indication for antibiotics that was most frequently missed was mechanical ventilation (MIV or NIV, 40% missed), followed by increase in sputum purulence combined with another cardinal symptom (23% missed). The average duration of antibiotic therapy was 7 days. Long-acting bronchodilators (LABA and/or LAMA, +/−ICS) had already been a part of the prescribed base medication before admission in 186 cases (82%) and they were part of the discharge medication in 196 cases (90%).

### 3.3. Follow-up and Prevention

The adherence to the GOLD recommendations for follow-up and preventive measures was evaluated only for the 219 cases that were discharged alive. The results are summarized in Table 4.

An early follow-up was arranged or recommended in 70% of the cases (35% each), while in 30% of the cases no follow-up arrangement or recommendation was done. “Follow-up arranged” included the situation if patients were discharged to another hospital or an inpatient rehabilitation institution. The GOLD guidelines do not specify the type of rehabilitation that is recommended. However, any type of rehabilitation was arranged in 48 cases (22%), distributed amongst inpatient rehabilitation (32/48, 67%), outpatient rehabilitation (3/48, 6%), and outpatient physiotherapy (13/48, 27%). Vaccination statuses were poorly documented with only 37 out of 219 cases for influenza (17%) and 16 out of the 196 recommended cases for pneumococcae (8%). Smoking cessation was verifiably recommended to 25 out of the 95 active smokers (26%).

### 3.4. Outcome

The GOLD therapeutic recommendations that showed poor adherence were initial treatment with SABA and the application of NIV when indicated. Associations of adherence to these recommendations with patient outcomes (length of hospital stay and in-hospital death) were analyzed. No significant differences were found between patients that were treated according to the guidelines, and those who were not. An overview of the results of the statistical analysis can be found in the Supplementary Materials (Table S5).

## 4. Discussion

Our evaluation of acute COPD exacerbation management in hospital care has three main findings:

- GOLD recommendations lack implementation with regard to all investigated fields: 1. anamnesis and documentation, 2. therapy, and 3. follow-up and prevention.
- Particular deficits were found in the documentation of exacerbation severity and GOLD risk group, performance of BGA, initial treatment with short-acting bronchodilators, and the application of NIV.
- The implementation of therapy with systemic corticosteroids and antibiotics shows an improving trend since the European COPD audit in 2011.

Our studied population consisted of 184 patients with a mean age of 75 and 53% males. This makes our population slightly older than the population studied in the Swiss COPD Audit in 2011 in 19 Swiss hospitals (mean age 70.7 years) [12], which only analyzed the data of patients hospitalized for at least twelve hours. Sex, smoking status, and comorbidity distributions of our cohort were rather similar to the Swiss data from the European COPD audit [12]. While the classification into GOLD risk groups A-D was not reported in the Swiss COPD Audit, the GOLD stages I-IV in our cohort were slightly less severe with 38% being in the mild to moderate stages (vs. 24% in the Swiss COPD Audit) [12].

The majority of patients in the study cohort presented at the ED on their own behalf. In general, patients’ individual stress and coping mechanisms are crucial factors in the decision of whether to present at an emergency department or not. This may have led to a heterogeneous study population with regard to symptoms, severity, and objective urgency. However, according to GOLD, the fact that they presented at an ED classifies the exacerbation as “severe” per se, irrelevant of clinical presentation and diagnostic measures. Additionally, in 92% of the cases the ED presentation led to hospitalization, which indicates a requirement for hospital care. GOLD reports a list with potential indications for hospitalization assessment, which includes severe symptoms, acute respiratory failure, onset of new physical signs, failure to respond to initial medical management, presence of serious comorbidities, and insufficient home support [5]. The National Institute for Health and Care Excellence (NICE) guidelines suggest a similar assessment. However, due to the lacking routine documentation of the reasons for hospitalization, adherence to these indications could not be evaluated in this study.

### 4.1. Anamnesis, Diagnostics, and Documentation

Overall documentation of GOLD classification was poor in our cohort: While the spirometry-based classification into GOLD stages I-IV was documented in 63%, the GOLD risk group based on symptoms and exacerbation history was only documented in 40% of the cases. Even though the documentation of neither GOLD stage nor GOLD risk group is directly recommended in the GOLD guidelines for the management of AECOPD, the assessment and knowledge of GOLD risk group is crucial for the guideline-conforming treatment of stable COPD, because recommendations have been given based on the GOLD ABCD classification since 2011 [5]. While in the investigated situation physicians may focus on the effective treatment of the acute exacerbation, their prescriptions, recommendations, and documentation upon discharge play a big role in the further treatment of the patient—and therefore their future exacerbation risk and symptom control. Previous studies have shown that adherence to the GOLD guidelines for the management of stable COPD is poor in the primary care sector, especially in GOLD risk groups A and B [18,19]. This is another argument for the regular revision of the patient’s base therapy during hospitalization, and therefore the assessment of GOLD risk group. The classification by lung function into GOLD stages I-IV was documented in 63%, and the actual results from the last spirometry in only 24% in our cohort. Spirometry is not recommended in the acute situation of an exacerbation. However, the regular performance of a spirometry is recommended once a year for all COPD patients [11], thus its results should always be documented for future reference or at least be recommended upon discharge.

An explicit recommendation from the 2019 GOLD report is the assessment of the exacerbation’s severity, by examination of changes in mental status, use of accessory respiratory muscles, and respiratory rate, as well as the performance of an arterial BGA [5,20]. These examinations allow the identification and classification of possible acute respiratory failure and the installation of an adequate treatment. While arterial BGA is the most accurate method to determine hypoxemia, hypercarbia, and pH—parameters that are also crucial for the assessment of NIV indication—venous BGA is a relatively reliable substitute for the measurement of pH and pCO2 [21,22]. For the measurement of respiratory rate being a quick, non-invasive, simple, and inexpensive procedure, the proportion of its performance in our cohort (73%) is considerably low. The examination of altered mental status and use of accessory respiratory muscles was documented in very few cases (6% and 7%, respectively). Since in all documented cases the respective clinical sign was present, we believe that the actual assessment was performed in much higher percentages, and the discrepancy is mainly due to a lack of documentation. An arterial BGA was performed in 63%, and a venous BGA in an additional 8%, which is too seldom in view of the above-described reasons.

Chest imaging (CT or radiography) was performed throughout, which allowed for the identification of potential infiltrates and other additional and differential diagnoses, like congestion.

While this audit only evaluated the adherence to the 2019 GOLD recommendations for the management of acute exacerbations, other procedures, that are not part of these guidelines by GOLD, may play an important role. Recent studies have shown that chronic comorbidities and their correct treatment play a crucial role in patient outcomes and prognosis in both stable COPD and acute exacerbations [23,24]. While GOLD has dedicated an entire chapter of their report to the management of comorbidities in the stable COPD state [5], Celli et al. recommend to always consider acute decompensations of concomitant diseases or acute events such as heart failure, pneumonia, or pulmonary embolism as contributors to the worsening respiratory symptoms [23].

### 4.2. Therapy

One of the main pillars of in-hospital AECOPD treatment is OS. It is recommended for all severe AECOPD to improve patients’ hypoxemia, with a target oxygen saturation of 88–92% [5]. While 68% of all patients in our cohort were supplemented with oxygen, all but one with an initial saturation of less than 88% received OS (98%), which is a satisfactory result. However, blood gases are recommended to be checked frequently under oxygen supplementation [5], which did not happen consistently; only 35% of the patients receiving oxygen supplementation had at least one follow-up arterial BGA during the entire hospitalization. This discrepancy has direct implications on the assessment of possible indications for ventilation (respiratory acidosis, persistent hypoxemia). Within the available documentation, we found 64 cases with a clear indication for NIV, of which only 26% were treated accordingly. Due to the above-described lack of follow-up BGAs, the number of NIV indications might even be underestimated. Thus, on the one hand, the indications for ventilation are not sufficiently assessed; on the other hand, recommendations regarding NIV are often not followed. These results are alarming, since there is evidence that a low-threshold application of NIV in mildly and moderately acidotic patients speeds up recovery and reduces the risk of need for invasive ventilation [25]. The underlying reasons for this discrepancy should be further investigated. Time pressure, shortage of intermediate or intensive care capacities, and financial issues, as well as a lack of physicians’ awareness or patients’ preferences are only some of the possible causes. The implementation of MIV, when indicated, has to be looked at from a different perspective: even though indications and recommendations are clear, patients’ and relatives’ wishes would play a crucial role, due to MIV’s invasiveness and possible complications. In our cohort, all two patients who required and agreed to receive MIV were treated accordingly. None of the patients received MIV without a clear indication.

The second main pillar of AECOPD therapy is pharmacological treatment. With dyspnea being one of the chief symptoms, mainly driven by bronchoconstriction [26], short-acting inhaled beta-2 agonists (SABA) are the recommended initial symptomatic therapy [5]. In our cohort, only half of the patients received this recommended treatment upon admission (52%). When taking into account that patients may have their own preferences, the initial application of SAMA could be considered a reasonable compromise. Furthermore, some patients had been pre-treated with SABA or SAMA by paramedics on their way to the hospital. Nevertheless, even the overall rate of patients receiving SABA or SAMA upon admission or pre-treated by paramedics is only 68%. Since increased dyspnea was present upon admission in 77% of the cases, at least 9% of patients were not given the recommended symptomatic therapy. This discrepancy was already observed in the Swiss data of the 2011 COPD audit, with SABA and/or SAMA having been administered in 75% of the cases [12].

Once the initial symptoms are under control, systemic corticosteroids are recommended to improve oxygenation and reduce inflammation [5]. Several studies have shown that systemic glucocorticoids have a positive impact on recovery time, lung function, oxygenation, and length of hospitalization in AECOPD [27,28,29]. The recommendation was well implemented in our cohort with 89% receiving systemic corticosteroids and a median treatment duration of 5 days and median dosage of 40 mg, as recommended. This is a substantial improvement since 2011, when only 77% of patients in the Swiss COPD audit had received systemic corticosteroids.

The GOLD guidelines for antibiotic treatment reflect the evidence that patients with all three cardinal symptoms (increase in dyspnea, increase in sputum volume, increase in sputum purulence) and patients with two cardinal symptoms, if increase in purulence is one of them, benefit from an antibiotic therapy [30,31]. Furthermore, antibiotics are recommended for mechanically ventilated patients [5]. Other indications, like elevated serum levels of C-reactive protein and procalcitonin, are discussed controversially. Since the Swiss national guidelines from 2018 recommend the application of antibiotics if the serum procalcitonin is greater than 0.25 ng/mL [10], this indication was also evaluated in this audit. In our cohort, antibiotics were administered in 127 cases (56%). The evaluation against the above-described indications revealed that there was no clear indication for antibiotics in a fifth of these cases. Furthermore, 14% of the patients with a clear indication for antibiotics did not receive it. These results show a considerable discrepancy in guideline-conforming antibiotic treatment. The proportion of cases with antibiotic treatment was higher in the Swiss COPD audit in 2011 (73%) than in our data (56%), which is a positive trend. Nevertheless, the inconsistent adherence to the recommended indications in our cohort seems to reflect the controversy in the topic. Further randomized controlled trials are needed to improve the existing evidence.

The GOLD recommendation to continue therapy with long-acting bronchodilators was well implemented: all patients who had already been under a LABA and/or LAMA regimen upon admission (82%) were discharged with a LABA or LAMA (with or without ICS) prescription. However, out of the 40 patients (18%) who had not been treated with a long-acting bronchodilator before and were discharged alive, only 10 patients were discharged with one. Considering the fact that long-acting bronchodilators significantly improve lung function, dyspnea, and health status, and reduce the risk for re-exacerbations [5,20], this leaves ample room for improvement. In addition, in patients who develop further exacerbations on a LABA/ICS combination base therapy, the escalation to a triple therapy (LABA + LAMA + ICS) is recommended [5]. The newest GOLD guidelines also suggest to consider triple therapy in patients with a high eosinophil count [32]. In our cohort, out of the 40 cases with LABA/ICS treatment upon admission, only 13 (33%) were discharged on a triple therapy regimen. In the light of the existing evidence about improved lung function and reduced exacerbation risk, this proportion should be substantially higher [32,33,34,35].

### 4.3. Follow-Up and Prevention

An early follow-up (within 4 weeks] represents an opportunity to review the patient’s discharge medication and compliance [11]. Gavish et al. showed that early follow-up visits are associated with a lower risk for exacerbation-related readmissions [36]. In our population, a follow-up was arranged upon discharge in 77 cases (35%) and advised in the discharge letter in another 76 cases (35%). In the remaining 30%, no follow-up plan was documented. While follow-up is a simple yet effective method to improve COPD prognosis, it strongly depends on several factors such as individual patients’ wishes, access to medical care and social support. However, there is evidence that patients with respiratory diseases are at particular risk for early rehospitalizations [37], which calls for a strong recommendation of an early follow-up in this patient group.

Similarly, rehabilitation after AECOPD can reduce readmission rates and mortality and improve quality of life [38,39]. Although it is only explicitly recommended for GOLD groups B, C, and D, we abstained from an evaluation of rehabilitation rates in this sub-group of our cohort due to the missing documentation of GOLD group classification in 64% [5]. When taking into account both inpatient and outpatient rehabilitation facilities, as well as outpatient physiotherapy, only 22% of the overall cases in our cohort were taking part in some sort of rehabilitation program. This is a clear decline compared to the Swiss data from the European COPD audit in 2011, where 40% of the patients took part in a pulmonary rehabilitation program [12]. Possible reasons for the further decline of an already insufficient implementation could be a lack of available places and lack of reimbursement by health insurances.

Vaccination status of the patients in our cohort was poorly documented. In most cases there was no information about the patient’s vaccination status (83% influenza, 91% pneumococci). Arguably, vaccinations and the documentation thereof are of secondary importance in the situation of an AECOPD and could be performed in a primary care setting. In addition, the lack of documentation is not indicative of how many of the patients had actually been vaccinated. However, the recommended vaccinations can help to prevent exacerbations, pneumonia, and death [40,41,42]. Thus, in patients that do not visit a general practitioner on a regular basis, a hospitalization is a good opportunity to assess the vaccination status and at least recommend it to those not vaccinated.

Smoking cessation is the most effective strategy for slowing down the progression of COPD and reducing mortality [43]. According to the GOLD recommendations, smoking cessation is a key intervention that should be encouraged at every possible opportunity [5]. In our cohort, the cessation advice was documented for 26% of the active smokers. While it may have been advised in more cases than those documented, it needs to be emphasized that there is evidence that even very brief counselling sessions can lead to a higher smoking cessation rate [44]. Additionally, assistance for smoking cessation, such as nicotine replacement therapy or pharmacological products should be discussed with active smokers [11].

### 4.4. Outcome

The analyzed relationship between guideline adherence and the patient outcomes length of stay and in-hospital death did not show any associations. It is important to note that these outcomes are highly influenced by many other factors, such as age, comorbidities, social situation, and exacerbation severity. Therefore, a comprehensive analysis of the effect of guideline non-adherence on patient outcome requires multivariable models that integrate potential confounders. Focusing on audit results, such an analysis would be of the scope of this article but should definitely be investigated further. While the evidence on the application of SABA as an initial symptomatic treatment is weak, previous studies found that NIV application could reduce the length of hospital stay and in-hospital mortality in certain patients with AECOPD [45,46].

### 4.5. Limitations

This retrospective study and clinical audit come with some limitations. We conducted a retrospective data analysis, which was based on the treating physicians’ and nurses’ documentation. In this design, data quality and results are largely dependent on complete and correct documentation. Missing data in parameters such as respiratory rate and use of respiratory muscles were rated as not assessed, even though there was a possibility that they had been assessed, but not documented. Therefore, guideline adherence with regard to diagnostic steps could have been underestimated. Furthermore, we limited our analysis of pharmacological therapy to bronchodilators, systemic corticosteroids, and antibiotics. The application of additional substances like mucolytic drugs was not evaluated. In addition, physicians’ decisions on AECOPD management depend on the patient’s individual situation, history, and wishes, which were not considered in this evaluation. Lastly, the important effects on patient reported outcomes were not evaluated, due to unavailability in clinical routine management.

## 5. Conclusions

Our study showed that the implementation of GOLD guidelines for the management of AECOPD showed substantial weaknesses in our hospital. In particular, these concerned the assessment of exacerbation severity, performance of BGA, initial treatment with short-acting bronchodilators, and the application of NIV when indicated. With regard to corticosteroid and antibiotic treatment, guideline implementation has improved in KSBL when compared to the Swiss data of the 2011 European COPD audit. However, the determination of indications for antibiotic treatment still needs to be improved. Since the European COPD audit in 2011 reported similar results, we hypothesize that these findings might be transferrable to other European hospitals to a certain extent. The results highlight the importance of regular internal evaluations in clinical settings, e.g., in the form of clinical audits, on the one hand, and the need for provisions to improve guideline implementation on the other hand. The publication of this data may help to create awareness for potential deviations from the guidelines and encourage other institutions to conduct a clinical audit. The causal relationships between guideline non-adherence and both objective and patient reported outcomes need to be investigated further.

## Figures and Tables

**Table 1:** Summary of GOLD recommendations for management of AECOPD.

GOLD Recommendation	Indication
Diagnostic steps	
- Assess severity - Assess respiratory rate. - Perform arterial blood gas analysis. - Assess use of accessory respiratory muscles. - Assess changes in mental status (confusion, lethargy, coma).	All AECOPD
Chest radiography	All severe, but not life-threatening AECOPD
Respiratory support	
Supplemental oxygen therapy	All severe AECOPD, target oxygen saturation 88–92%
Non-invasive ventilation (NIV)	- Respiratory acidosis. - Severe dyspnea with clinical signs suggestive of respiratory muscle fatigue, increased work of breathing, or both. - Persistent hypoxemia despite supplemental oxygen therapy.
Mechanical invasive ventilation (MIV)	- Unable to tolerate NIV, or NIV failure. - Status post respiratory or cardiac arrest. - Diminished consciousness, psychomotor agitation inadequately controlled by sedation. - Massive aspiration or persistent vomiting. - Persistent inability to remove respiratory secretions. - Severe hemodynamic instability without response to fluids and vasoactive drugs. - Severe ventricular or supraventricular arrhythmias. - Life-threatening hypoxemia in patients unable to tolerate NIV.
Pharmacological therapy	
Short-acting beta-2 agonists (SABA)	All AECOPD, initial symptomatic treatment
Systemic glucocorticoids (40 mg prednisone, no longer than 5–7 days)	All severe AECOPD
Antibiotics (5–7 days) ^a^	Patients with three cardinal symptoms: Increase in dyspnea. Increase in sputum volume. Increase in sputum purulence. Patients with increased purulence, plus at least one other cardinal symptom. Patients who require mechanical ventilation.
Long-acting bronchodilators (LABA or LAMA) +/−ICS	All AECOPD, continue or start before hospital discharge
Follow-up and Prevention	
Clinical follow-up within 4 weeks	All AECOPD
Clinical follow-up within 3 months, incl. spirometry ^b^	All AECOPD
(Pulmonary) rehabilitation	GOLD risk group B, C, and D
Smoking cessation	Current smokers
Physical activity	All COPD patients
Vaccination - Influenza - Pneumococcal	- Influenza: all COPD patients - Pneumococcal: in patients ≥65 years old, or FEV1 <40% predicted, or with significant comorbidities

GOLD: Global Initiative for Chronic Obstructive Lung Disease; AECOPD: Acute exacerbation of chronic obstructive pulmonary disease; NIV: non-invasive ventilation; MIV: mechanical invasive ventilation; FEV1: Forced expiratory volume in one second; ^a^ indication for antibiotic treatment was additionally checked against the serum procalcitonin-guided approach recommended in the 2018 Swiss national guidelines [10]; ^b^ compliance with the 3-month follow-up recommendation was not evaluated, because follow-up was mainly organized in a decentralized manner.

**Table 2:** Anamnesis, diagnostics and documentation upon admission.

Procedure/Documentation	Performed, *n* (%)
Cases Overall	*n* = 226
*Anamnesis and documentation*	
GOLD risk group (A–D) ^a^	91 (40)
Smoking status ^a^	206 (91)
*Vital signs and clinical examination*	
Respiratory rate	164 (73)
Assessment of changes in mental status	14 (6)
Assessment of use of respiratory muscles	15 (7)
Oxygen saturation	220 (97)
*Laboratory diagnostics*	
Arterial blood gas analysis	141 (62)
*Imaging and lung function testing*	
Chest radiography	212 (94)
Results of last spirometry documented ^a^	54 (24)

GOLD: Global Initiative for Chronic Obstructive Lung Disease; ^a^ not directly recommended but needed for the implementation of other GOLD recommendations.

**Table 3:** Treatment of AECOPD in emergency department and during hospitalization.

Therapy	Administered, *n* (%)
Cases Overall	*n* = 226
*Respiratory Support*	
Supplemental oxygen in cases with oxygen saturation <88%	47/48 (98)
Non-invasive ventilation in case of a clear indication	15/64 (23)
Invasive mechanical ventilation in case of a clear indication	2/7 (29)
*Pharmacological Therapy*	
Initial symptomatic therapy with SABA (with or without SAMA)	118 (52)
Systemic steroids	200 (89)
Duration of systemic steroids, days (median [IQR])	5 [5,6]
Systemic steroids for ≤7 days	180/189 (95)
Systemic steroids daily dosage 40 mg prednisone equivalent	143/188 (76)
Antibiotics	127 (56)
Duration of antibiotics, days (median [IQR])	7 [6,9]
Antibiotics administered, without clear indication ^a^	27/127 (21)
No antibiotics administered in case of a clear indication ^a^	16/116 (14)
Long-acting bronchodilators +/− ICS at discharge (if discharged alive, *n* = 219)	196 (90)

AECOPD: acute exacerbation of chronic obstructive pulmonary disease; IQR: interquartile range; SABA: short-acting beta-2 agonist; SAMA: short-acting antimuscarinic antagonist; ICS: inhaled corticosteroid; ^a^ apart from indications listed in Table 1, a procalcitonin-guided regimen was also considered a clear indication [10].

**Table 4:** Adherence to the GOLD guidelines regarding follow-up and prevention for AECOPD.

GOLD Recommendation	Performed, *n* (%)
Cases Overall	*n* = 219
*Follow-up*	
Arranged	77 (35)
Advised	76 (35)
*Rehabilitation*	
Any type of rehabilitation arranged	48 (22)
*Vaccination Influenza*	
Status active or performed during hospitalization	18 (8)
Advised	19 (9)
Not documented	182 (83)
*Vaccination Pneumococcal (n = 196) ^a^*	
Status active or performed during hospitalization	4/196 (2)
Advised	12/196 (6)
Not documented	178/196 (92)
*Smoking cessation, recommended for active smokers (n = 95)*	
Advised	25/95 (26)

GOLD: Global Initiative for Chronic Obstructive Lung Disease; FEV1: Forced expiratory volume in one second. ^a^ recommended in patients ≥ 65 years old, or FEV1 < 40%, or with significant comorbidities.

## Data Availability

The data presented in this study are available from the corresponding author upon reasonable request. The data is not publicly available due to restrictions in data privacy.

## Supplementary Materials

The following supporting information can be downloaded at: https://www.mdpi.com/article/10.3390/jcm13010019/s1, Figure S1. Case selection process; Table S1. Patient and case characteristics; Table S2. Additional anamnesis, diagnostics, and documentation upon admission; Table S3. Additional treatment of AECOPD in emergency department and during hospitalization; Table S4. Details on rehabilitation arrangements; Table S5. Association of guideline adherence with length of hospital stay and in-hospital mortality.
