# Supplementary material for: The Management of Acute Exacerbations in COPD: A Retrospective Observational Study and Clinical Audit
Source: J Clin Med. 2023 Dec 19;13(1):19. doi: 10.3390/jcm13010019 (PMC10779377; doi:10.3390/jcm13010019)
Supplement: Supplementary file 1 [file jcm-13-00019-s001.zip › jcm-2723105-supplementary.pdf]

Management of Acute Exacerbations in COPD: A Retrospective Observational Study and Clinical Audit

Supplementary Material

Results

1. *Patient and case characteristics*

Figure S1 presents a flow chart of the case selection process. Cohort characteristics at patient level and case level are presented in Table S1.

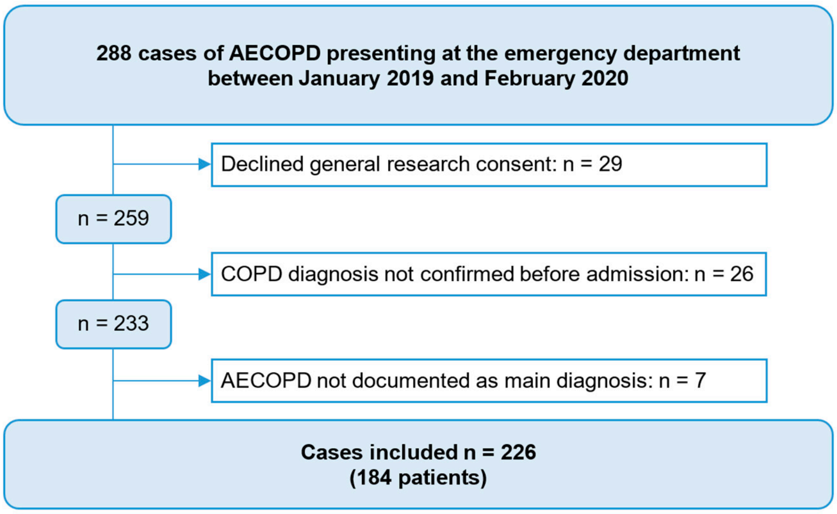

Figure S1. Case selection process. AECOPD: Acute exacerbation of chronic obstructive pulmonary disease. COPD: Chronic obstructive pulmonary disease.

All but two patients (99.5%) suffered from at least one chronic comorbidity, with cardiovascular diseases being the most common. More than half of the patients suffered from hypertension (n = 104, 57%), and around a quarter from ischemic heart disease (n = 49, 27%). Other frequent comorbidities were diabetes mellitus, an asthma overlap, arrhythmias, malignant diseases, peripheral arterial diseases, and depression.

The main symptoms leading to the emergency consultation were the cardinal symptoms of AECOPD (increased dyspnea, increased cough, increased sputum volume, and increased sputum purulence/change in color, see Table 2). In approximately 20% of the cases (n = 49), other symptoms like fever, decreased oxygen saturation, body aches, or general deterioration were mentioned.

In almost 40% (n = 87) exacerbation treatment had already been initiated before admission, by either a general practitioner or the referring doctor or the paramedics. Of these, approximately 52% (45/87) received SAMA and/or SABA from the paramedics, and around 45% had already been administered an oral corticosteroid (40/87) and/or antibiotics (41/87). In one case, the patient was admitted already intubated and in three other cases (1%), the patient had already received NIV from the paramedics.

The most common pre-existing base medication regimen was LAMA+LABA+ICS (n = 77, 34%), followed by the combination of LAMA and LABA (n = 61, 27%). Concerning short-acting bronchodilators, the most common medication was a SAMA+SABA combination (n = 35, 16%). In 24% of the cases (n = 54) home oxygen therapy was implemented. In 10% percent of the cases (n = 23) no specific COPD therapy had been prescribed.

Table S1. Patient and case characteristics. Data presented as n (%) if not otherwise stated.

**Patients overall**	**n = 184**	**Missing data**
Demographics and BMI		
Age, years (median [IQR]) *(range)*	75 [67, 79] *(41 - 95)*	0
Sex, male	97 (53)	0
Smoking status		18 (10)
Current smoker	81 (49)	
Former smoker	82 (49)	
Lifelong non-smoker	3 (2)	
Pack years (median [IQR])	50 [40, 65]	42 (23)
GOLD stage		72 (39)
I	10 (9)	
II	34 (29)	
III	39 (35)	
IV	29 (26)	
GOLD risk group		118 (64)
A	3 (5)	
B	17 (26)	
C	13 (20)	
D	33 (50)	
Comorbidities		0
Cardiovascular disease	143 (78)	
Diabetes mellitus	37 (20)	
Asthma overlap	25 (14)	
**Cases overall**	**n = 226**	
Hospitalization		0
Hospitalized, normal ward	183 (81)	
Hospitalized, IMC	4 (2)	
Hospitalized, ICU	21 (9)	
Outpatient	18 (8)	
Symptoms		0
Increased cough	111 (49)	
Increased dyspnea	175 (77)	
Increased sputum volume	13 (6)	
Sputum color changed	55 (24)	
Clinical examination		0
Cyanosis	6 (3)	
Use of accessory respiratory muscles	15 (7)	
Edema	32 (14)	
Confusion	14 (6)	
COPD base therapy before admission		0
SAMA	12 (5)	
SABA	31 (14)	
SAMA + SABA	35 (16)	
LAMA	10 (4)	
LABA	3 (1)	
ICS	3 (1)	
LABA + ICS	40 (18)	
LABA + LAMA	61 (27)	
LABA + LAMA + ICS	77 (34)	
oral theophylline	2 (1)	
oral steroid	9 (4)	
oxygen home therapy	54 (24)	
others	11 (5)	
none	23 (10)	

GOLD: Global Initiative for Chronic Obstructive Lung Disease; BMI: body mass index; IQR: interquartile range; IMC: Intermediate care unit; ICU: intensive care unit; SAMA: short-acting antimuscarinic antagonist; SABA: short-acting beta-2 agonist; LABA: long-acting beta-2 agonist; LAMA: long-acting muscarinic antagonist; ICS: inhaled corticosteroid.

2. *Anamnesis, Diagnostics, and Documentation*

Table S2 presents additional anamnesis, diagnostics, and documentation, supplementary to Table 2.

Table S2. Additional anamnesis, diagnostics, and documentation upon admission.

Procedure / Documentation	Performed, n (%)
Cases overall	n = 226
Anamnesis and documentation	
GOLD stage (I-IV)	143 (63)
Vital signs and clinical examination	
Body temperature	223 (99)
Laboratory diagnostics	
Venous blood gas analysis	17 (8)
C-reactive protein	225 (100)
Leucocytes	224 (99)
Procalcitonin	66 (29)
Eosinophil granulocytes	208 (92)
Chest radiography	
X-Ray	182 (81)
Computer tomography	16 (7)
Both	14 (6)
Lung function	
Spirometry (at any time during hospitalization)	23 (10)

GOLD: Global Initiative for Chronic Obstructive Lung Disease

3. *Therapy*

Table S3 presents additional treatment supplementary to Table 3.

Table S3. Additional treatment of AECOPD in emergency department and during hospitalization.

Therapy	Administered, n (%)
**Cases overall**	**n = 226**
Respiratory Support	
Any form of supplemental oxygen	154 (68)
Nasal prongs or mask	134/154 (87)
High-flow nasal cannula	0 (0)
Non-invasive ventilation (NIV), overall	18/154 (12)
Time-to-oxygen, minutes (median [IQR])	5 [0,15]
Pharmacological Therapy	
Initial symptomatic therapy	
SAMA only	14 (6)
Time-to-SABD, minutes (median [IQR])	45 [20, 90]
Further medication	
Daily dosage systemic steroids,	
mg prednisone equivalent (median [IQR]) (n = 188)	40 [40,40]
Antibiotics	127 (56)

AECOPD: acute exacerbation of chronic obstructive pulmonary disease; NIV: non-invasive ventilation; IQR: interquartile range; SAMA: short-acting antimuscarinic antagonist; SABD: short-acting bronchodilator.

4. *Follow-up and prevention*

Table S4 presents details with regard to rehabilitation arrangements supplementary to Table 4.

Table S4. Details on rehabilitation arrangements.

GOLD recommendation	Performed, n (%)
**Cases overall**	**n = 219**
Rehabilitation	
Inpatient rehabilitation	32/48 (67)
Outpatient rehabilitation	3/48 (6)
Outpatient physiotherapy	13/48 (27)

GOLD: Global Initiative for Chronic Obstructive Lung Disease

5. *Outcome*

Table S5 presents the comparison of the outcomes of patients treated according to the guidelines vs. patients not treated according to the guidelines with regard to initial treatment with SABA and application of NIV if indicated.

Table S5. Association of guideline adherence with length of hospital stay and in-hospital mortality.

	Initial treatment with SABA (n = 226)			NIV if indicated (n = 64)		
**Outcome**	**Yes**	**No**	**p-value**	**Yes**	**No**	**p-value**
Length of hospital stay[a], median [IQR]	8 [6,11]	7 [5,11]	0.098	9 [7,12]	8.5 [6,11]	0.74
In-hospital death, n (%)	3 (2.5)	4 (3.7)	0.905	3 (6.8)	3 (17.6)	0.427

[a] only hospitalized patients (n = 208); SABA: short-acting inhaled beta-2 agonists; NIV: non-invasive ventilation; IQR: Interquartile range.
